# Immune Checkpoints in Leprosy: Immunotherapy As a Feasible Approach to Control Disease Progression

**DOI:** 10.3389/fimmu.2017.01724

**Published:** 2017-12-11

**Authors:** Hayana Ramos Lima, Thaís Helena Gasparoto, Tatiana Salles de Souza Malaspina, Vinícius Rizzo Marques, Marina Jurado Vicente, Elaine Camarinha Marcos, Fabiana Corvolo Souza, Maria Renata Sales Nogueira, Jaison Antônio Barreto, Gustavo Pompermaier Garlet, João Santana da Silva, Vânia Nieto Brito-de-Souza, Ana Paula Campanelli

**Affiliations:** ^1^Department of Biological Sciences, Bauru School of Dentistry, University of São Paulo, Bauru, Brazil; ^2^Lauro de Souza Lima Institute, Bauru, Brazil; ^3^Department of Biochemistry and Immunology, School of Medicine of Ribeirão Preto, University of São Paulo, Ribeirão Preto, Brazil

**Keywords:** immunotherapy, leprosy, T-regulatory cells, immune checkpoint blockade, PD-1:PD-L1, cytotoxic T-lymphocyte-associated protein 4

## Abstract

Leprosy remains a health problem in several countries. Current management of patients with leprosy is complex and requires multidrug therapy. Nonetheless, antibiotic treatment is insufficient to prevent nerve disabilities and control *Mycobacterium leprae*. Successful infectious disease treatment demands an understanding of the host immune response against a pathogen. Immune-based therapy is an effective treatment option for malignancies and infectious diseases. A promising therapeutic approach to improve the clinical outcome of malignancies is the blockade of immune checkpoints. Immune checkpoints refer to a wide range of inhibitory or regulatory pathways that are critical for maintaining self-tolerance and modulating the immune response. Programmed cell-death protein-1 (PD-1), programmed cell death ligand-1 (PD-L1), cytotoxic T-lymphocyte-associated protein 4, and lymphocyte-activation gene-3 are the most important immune checkpoint molecules. Several pathogens, including *M. leprae*, are supposed to utilize these mechanisms to evade the host immune response. Regulatory T cells and expression of co-inhibitory molecules on lymphocytes induce specific T-cell anergy/exhaustion, leading to disseminated and progressive disease. From this perspective, we outline how the co-inhibitory molecules PD-1, PD-L1, and Th1/Th17 versus Th2/Treg cells are balanced, how antigen-presenting cell maturation acts at different levels to inhibit T cells and modulate the development of leprosy, and how new interventions interfere with leprosy development.

## Introduction

Leprosy remains a relevant health problem in Brazil and India even after the introduction of multidrug therapy and has spread worldwide (1–3). Leprosy presents different clinical features that are determined by the host immune response against *Mycobacterium leprae*; at the pole of this spectrum are tuberculoid and lepromatous disease. In tuberculoid leprosy (TT), Th1 polarization, characterized by the production of IFN-γ, which activates CD8 T cells, macrophages and bactericidal mechanisms that control *M. leprae* growth, is critical for the protective response (2, 4, 5). By contrast, lepromatous leprosy (LL) presents with impaired specific cellular immunity. The immune response often differentiates into a Th2 profile, with abundant production of IL-4 and predominant B cell activation, which allows for evasion by the bacillus. *M. leprae* shows strategies to limit the host protective immune response leading to chronic infection (6, 7). In chronic infections, T cells are exposed to persistent antigen stimulation as a gradual loss of effector functions and cytokine production as well as persistently increased expression of multiple inhibitory receptors (6, 8). The immunomodulatory properties from mycobacteria have been explored to understand macrophage function (5, 9). In addition, *M. leprae* antigens interfere with T-cell proliferation (10) and are involved in Treg-cell expansion through HSP-60 (11). Evidence has indicated that Treg cells, besides expression of immune checkpoint molecules with inhibitory activity, such as PD-1, PD-L1, and cytotoxic T-lymphocyte-associated protein 4 (CTLA-4), induce specific T-cell anergy, leading to disseminated and progressive disease (7, 12, 13). Immune checkpoint (ICP) molecules play an important role in T-cell activation and determine the functional outcome of T cells, reducing the proliferation and secretion of inflammatory cytokines, such as IL-2, IFN-γ, and TNF-α (14, 15). Those molecules also interfere with dendritic cell (DC) maturation and macrophage effector function (5, 16). ICP, particularly PD-1/PD-L1 and CTLA-4, have been widely explored as therapeutic targets in cancer because these biomarkers are also highly expressed in the tumor microenvironment (14, 15). In infectious diseases, this therapeutic approach has been applied against HIV, HCV, and tuberculosis as an adjuvant of antimicrobial drugs (17–19).

Herein, to discuss new approaches for leprosy monitoring and treatment, we reviewed some of the ICP for leprosy persistence and mechanisms associated with T-cell lymphocyte anergy to *M. leprae* antigens as well as the role of Treg cells to modulate disease development.

## Immune Checkpoints in Leprosy

Although ICP have been studied for approximately two decades, many features of their biology and signaling pathways remain unknown. ICP receptors are associated with autoimmunity, suggesting that these molecules play a critical role in immune tolerance and homeostasis (7, 8). In chronic infections, T lymphocytes are under persistent exposure to antigens, and this stimulus is commonly associated with T exhaustion (20). Various ICP molecules are highly expressed on exhausted T cells (14, 20), and this literature indicates that ICP blockade can restore immunity after reversion of the exhaustion phenotype of T cells (8). In leprosy, some recent data have shown a strict relationship between ICP expression and disease persistence.

**Figure 1:**
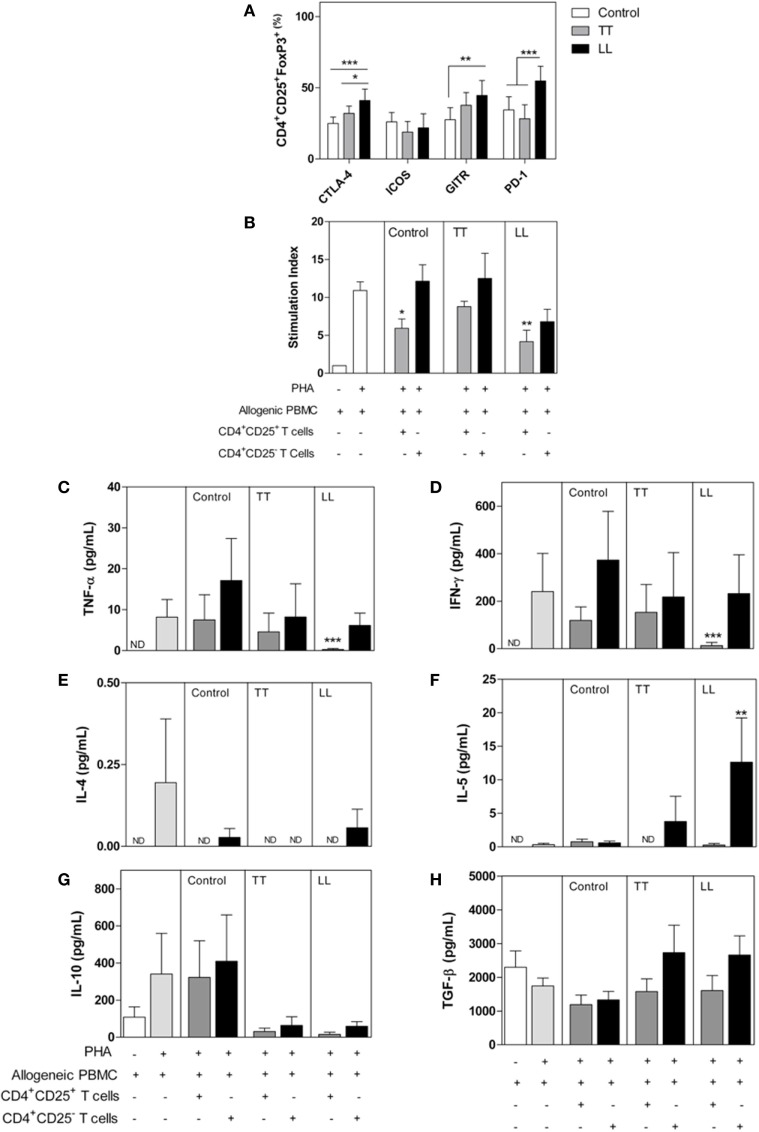
Phenotype and functional characterization of CD4^+^CD25^+^ T cells in leprosy patients. Peripheral blood mononuclear cells (PBMCs) were isolated from patients with tuberculoid (TT, *n* = 12) and lepromatous leprosy (LL, *n* = 12), as well as from healthy control subjects (*n* = 12). **(A)** The frequency of CD25^+^ and FoxP3^+^ cells and expression of cytotoxic T-lymphocyte-associated protein 4 (CTLA-4), GITR, ICOS, and PD-1 were determined by flow cytometry. **(B)** Allogeneic PBMC (1 × 10^5^ cells/well) was cultured with medium only, PHA, PHA plus CD4^+^CD25^+^ T, or CD4^+^CD25^−^ T cells (1 × 10^4^ cells/well) from patients or control subjects. Proliferation was determined after 4 days of culture by CFSE dilution analyzed by flow cytometry. The results are expressed as the means ± SEM of the stimulation index of proliferation. IFN-γ **(C)**, TNF-α **(D)**, IL-4 **(E)**, IL-5 **(F)**, IL-10 **(G)**, and TGF-β **(H)** levels were determined in supernatants from cultures of suppression assays. The results are presented as the means ± SEM. *p < 0.05, **p < 0.01, and ***p < 0.001, compared with control subjects using ANOVA and the Bonferroni posttest. For the suppressive assay **(B)**, the results are expressed as the means ± SEM; *p < 0.05, **p < 0.01, and ***p < 0.001, compared with the proliferation of allogeneic PBMCs cultured with PHA. ND, not detected.

Cytotoxic T-lymphocyte-associated protein 4 is an important molecule that controls lymphocyte activation (21). This molecule binds to CD80/CD86, antagonizing CD28 signaling, on antigen-presenting cell (APC) cells, leading CD4^+^ and CD8^+^ T cells to assume an anergic phenotype (14, 22). Some CTLA-4 signaling pathways are still unknown, and it is unclear how this receptor interferes with lymphocyte activation as well as how CD3 phosphorylation, ZAP-70 activation, or tyrosine phosphatase SHP-2 act as intracellular mediators of those pathways (21). Indeed, CTLA-4 is essential for Tregs function. Treg cells highly express CTLA-4, which controls DC maturation, leading to internalization of CD80 and/or CD86 in addition to indoleamine-2,3-dioxygenase (IDO) activation, leading to expression of the immunosuppressive mediator kynurenin (16, 21, 23). These signals can also promote nuclear localization of Foxo, a transcriptional factor that suppresses transcription of the genes encoding IL-6 and TNF-α, both of which are crucial effector cytokines for the control *M. leprae* infection (6, 22). In LL patients, CTLA-4 has been described as a biomarker in blood and inflammatory infiltrating cells (24, 25). Increased expression of CTLA-4 was detected in LL lesions compared with that in TT lesions (24). Our group has found increased expression of CTLA-4 on lymphocytes and Treg cells from LL patients in contrast to reduced CTLA-4 expression on the same cell populations of TT patients^1^ (Figure 1). We also observed that CD4^+^CD25^−^ T cells obtained from LL patients suppressed allogenic proliferation in functional tests (Figure 1). A suppressive role of CTLA-4 has also been demonstrated in FoxP3^−^ T cells, and these data might explain the suppressive profile presented by LL patients (26). We also observed that CD4^+^CD25^−^ T cells obtained from LL patients suppressed allogenic proliferation on functional tests (Figure 1). In TT patients, *in vitro* blockade of CTLA-4 restored peripheral blood mononuclear cell (PBMC) proliferation (12), but there is no clinical trial showing those effects on LL patients. Immunotherapy (IT) with CTLA-4 blockade has mostly been conducted against tumoral cells; nonetheless, recent evidence has shown that CTLA-4 expression is associated with reduced secretion of TNF-α and IFN-γ and enhanced frequency of memory CD8^+^ lymphocytes in experimental *L. monocytogenes* infection (27). Similarly, CTLA-4 blockade induced higher production of IFN-γ and NO when T cells were stimulated with *Trypanosoma cruzi* antigens, although it did not restore lymphocyte proliferation (28). Furthermore, despite few clinical trials concerning CTLA-4 blockade to control infectious diseases, HCV patients demonstrated promising results after this therapy (29). Taken together, these data suggest that immunotherapies might modulate the immune system in patients with a latent leprosy infection or active disease, enabling better control of *M. leprae* replication. Therefore, new discoveries concerning the role of CTLA-4 in the immune response during *M. leprae* infection could provide critical insight that can be applied to other infectious diseases.

Programmed cell-death protein-1 (PD-1) and its ligands PD-L1/L2 have also been identified as relevant ICP that promote immune evasion of tumor cells and infected cells (8, 14, 15). Those molecules are promising targets in anticancer therapy and are implicated in dysfunctional acquired immune responses, reducing the TCR signal to lymphocyte proliferation through ITIM (immune receptor tyrosine-based inhibition) motifs (30). The PD-1 signaling axis has been strongly related to T-cell anergy, pathogen persistence, and peripheral immune tolerance (14, 30). Although not yet targeted clinically, PD-1 is a promising target for leprosy IT. In leprosy, patients have presented with increased expression of PD-1 and PD-L1 on CD4^+^, B cells, and CD11^+^ cells (12, 13, 24, 31, 32), and *in vitro* blockade of PD-1 increased IFN-γ and IL-17 production by T cells (33). In accordance, our group found increased expression of PD-1 and GITR on lymphocytes and Tregs from LL patients (Figure 1). Blockade of PD-1 signaling in infectious disease has been associated with pathogen control in animal models for HBV, HIV, *Plasmodium* spp., *Leishmania* spp., *Trypanossoma* spp., and *M. tuberculosis* infection (34–38). These results suggest that ICP might be an important mechanism to regulate the immune response of LL patients. Thus, antibodies targeting the PD-1 pathways may improve the clinical outcome by restoring T-cell-mediated *M. leprae* immunity. However, in the infectious diseases context, immunotherapies based on ICP have not been tested or developed to the same extent as they have in cancer (39).

More recently, the signaling pathways and inhibitory mechanisms of lymphocyte-activation gene-3 (LAG-3) and TIGIT (T-cell immunoglobulin and ITIM domain) have also been explored as suitable new targets for immune blockade (14, 40, 41). TIGIT, a member of the CD28 family, is expressed on effector and memory T cells, Tregs, and natural killer (NK) cells, and its ligands, CD155 and CD122, are expressed on APC, T cells, and non-hematopoietic cell types, such as tumor cells (14, 40). TIGIT blockade might influence both adaptive and innate immune responses. TIGIT^+^ Treg cells seem to control the Th1/Th17 ratio through enhanced IL-10 secretion, leading to a Th2 phenotype in animal models (42). In addition, TIGIT also controls NK cell function, limiting IFN-γ secretion and granule production (43–45); however, there are no available data concerning the role of this molecule on NK cells during leprosy. In HIV-infected subjects, PD-1, TIGIT and LAG-3 are considered to be biomarkers of persistent infection because CD4^+^ T cells expressing these molecules present viral markers, even under antiretroviral therapy (41). Analysis of TIGIT expression on Th2 lymphocytes and Tregs from LL patients as well as its correlation with disease progression are highly warranted.

Regarding LAG-3, this molecule is also expressed on Treg cells and has been associated with increased suppressive events, such as in the immune response against HIV and *Plasmodium* spp., as well as many types of cancers (14, 41, 46). In leprosy patients, the role of LAG-3 remains unknown, although LAG-3^+^CD8^+^ T cells were detected when human PBMC cells were cultured with *M. leprae* as well as in human mycobacteria-induced granulomas (47). Some clinical trials have explored LAG-3 blockade to achieve tumor reduction and control cancer progress. Although the LAG-3 signaling pathway is not completely understood in *M. leprae* immunity, its homology to CD4 and cross-linking with MHC class II lead to impaired maturation of DC and Treg development (48, 49), suggesting LAG-3 as a new target for therapeutic intervention. Therefore, new discoveries concerning the role of this molecule in the immune response during *M. leprae* infection could provide insights that can be applied to other infectious diseases.

Recently, some evidence has indicated that combined ICP blockade might be a better strategy to explore the synergic effect of multiple immune checkpoints. Single-agent ICP approaches seem to induce compensatory upregulation of other ICPs as a cell mechanism to evade IT effects, and its failure index has been observed in one-half of oncology patients under this therapy (50, 51). One possibility would be to associate independent and non-redundant inhibitory pathways of these molecules, as observed in the CTLA-4 or PD-1 combination (50, 51). In addition, each infectious disease has its own ICP pattern of expression, as observed in HBV patients whose PD-1 expression is higher than that of CTLA-4. Therefore, combined ICP blockade might be a relevant mechanism for immune response regulation in leprosy and, likely, a feasible pathway to be explored as therapeutic targets.

## T Regulatory Cells (Tregs)

T regulatory cells (Tregs) are a heterogeneous subset of T CD4^+^ lymphocytes that might be developed at the thymus or on peripheral tissues under the control of many different signals from the microenvironment, such as TGF-β and IL-10 cytokines, retinol, and pathogen-associated molecular patterns (52, 53). Treg cells control many innate and adaptative immunological events, limiting tissue damage and maintaining homeostasis. Tregs explore many different mechanisms that control the immune response, such as increasing expression of CD25, to reduce lymphocyte proliferation through IL-2, increasing secretion of anti-inflammatory cytokines and increasing expression of granzyme and perforin, as well as ICPs, such as PD-1, CTLA-4, GITR, TIGIT, and LAG-3 (14, 40, 52–54). Treg cells in the infection site seems to be associated with the immune hyporesponsiveness observed after infection with many parasites, including *T. cruzi, P. brasiliensis, L. brasiliensis*, and *S. mansoni* (52–58). Some studies have shown the potential of Treg-cell depletion to augment antitumor immune responses (59) and infectious disease outcome (60) and have indicated that Treg-mediated T-cell suppression is an important mechanism by which pathogens evade immune responses (39, 53). In leprosy, an increased frequency of Treg cells was observed, and those lymphocyte subsets seem to contribute to pathogen persistence (13, 31–33, 61, 62). Our group assessed the suppressor features of Treg cells isolated from leprosy patients (Figure 1). Functional suppressive assays demonstrated impaired proliferation of allogenic PBMCs that were CFSE-labeled when cocultured with CD25^+^ T cells isolated from LL patients. In addition, the IFN-γ and TNF-α production levels were reduced in the presence of CD4^+^CD25^+^ T cells from LL patients. Moreover, our results showed that Treg cells (Foxp3^+^CD25^+^ cells) express high levels of CTLA-4 and PD-1. Such regulatory features were not hallmarks of Treg cells from TT patients (Figure 1). Expression of CTLA-4 by Treg cells serves as a mechanism of Treg cells to suppress excessive T-cell responses. Blocking CTLA-4 *in vivo* has been shown to inhibit Treg cells and promote antitumor immunity (63).

Activated Tregs produce IL-10, IL-35, and TGF-β, which act to suppress the immune response (64). Tregs downregulate the immune reactions through production of anti-inflammatory cytokines, lowering the antigen-presenting function in DCs, and macrophages with correspondingly decreased counts of Th1, Th2, Th17 CD4^+^ T cells, and cytotoxic CD8^+^ T cells, as well as the cytokines produced by them, and induction of apoptosis [reviewed in Ref. (65)]. High levels of TGF-β and IL-10 producing Foxp3^+^ T cells were reported to be increased in the lepromatous state in the circulation and skin lesions (61). Recent work has demonstrated that Tregs play a role in *M. leprae*-specific Th1 unresponsiveness during lepromatous disease (33). In LL, Th2/Treg polarization seems to be important to disease progression, and multiple factors may be responsible for these events, such as antigen exposure and innate immune activation (7). Otherwise, TT patients present a cellular immune response polarized to Th1/Th17 (31–33). Recent work has shown that in patients with a type 2 reaction, downmodulation of Tregs favors the development of Th17 responses (66, 67). The FoxP3^+^ Treg phenotype seems to be reverted into Th17, exploring the signaling through IL-12 and IL-23 (31). Th17 and Treg cells are new players associated with immunopathology in leprosy and its reactions (62). In this context, the ideal treatment for LL patients seems to require modulation of the T lymphocytes subsets to expand Th17 lymphocytes and control Treg cells, favoring the cellular immune response (5, 33). This strategy to shift the immune response to Th1/Th17 probably might achieve better outcomes in leprosy treatment; however, it might be associated with an increased risk of developing reactional states, such as erythema nodosum (66, 67). Reactional episodes have been associated with immune stimulation and can occur at any moment during leprosy infection and represent one of the most adverse events associated with disease (2, 5). Future work will need to confirm the efficacy of Treg cells for IT of infectious diseases. In addition, it is important to analyze the combinations of Treg cell targeting with ICP blockade to make IT more effective.

## Antigen-Presenting Cells

Because leprosy is an intracellular infection, T-cell activation and responses are important for protective immunity. It is well known that macrophages and DCs regulate the activity of lymphocytes in adaptive immune responses, which could allow them to play important roles in IT (68). This capacity makes them potent adjuvants for the induction of antigen-specific T cells in infected hosts. In leprosy, data have shown that a delicate balance of costimulatory pathways between T-cell and APCs is essential for T-cell activation.

Dendritic cells play important roles in both innate and acquired immunity responses to *M. leprae* infection. These cells induce Th1 immunity and CTL responses (2, 69). However, *M. leprae* have evolved mechanisms to inhibit the ability of DCs to present antigens, thereby promoting a protective immune response. Exposure of DCs to *M. leprae* impairs its maturation and inhibits CD80, CD86, HLA-DR and CD40 expression (70–72). Recognition of *M. leprae* antigens, such as LAM, through DC-SIGN has also been described as an important signaling pathway to control DC maturation, leading to increased IL-10 secretion, increased lipid metabolism and bacterial persistence (73, 74). Furthermore, IDO is another molecule associated with DC maturation and its tolerogenic phenotype. IDO has also been detected at high levels in LL patients (75). IDO catalyzes the conversion of tryptophan to *N*-formyl-kynurenine, and this molecular messenger controls cell proliferation, induces apoptosis, and shifts T-naïve cells to develop into Tregs (52, 75). DC-based IT has been used to improve the immune response against tumor cells using DC vaccines and blocking ICP associated with DC tolerogenic phenotypes (76). To improve the immune response in chronic infectious diseases, such as HBV infection, PD-L1 blockade has been used to restore the production of Th1 cytokines, such as TNF-α, IL-2, and IFN-γ (34). On the other hand, DC vaccines have also been used to improve the immune response to the Th17 profile against *Leishmania* spp. infection (77). Application of DCs in IT against *M. leprae* has not been explored despite the potential for the stimulation of an efficient antibacterial immunity. In other disease, the results indicate that DC-based IT might be more effective in combination with conventional treatments because the association should modulate the immune system in a way that helps the host control or eliminate pathogens (68, 77, 78). Therefore, exploring strategies to shift the immune response to Th1 might achieve better outcomes in leprosy treatment, leading to reduced expression of *M. leprae* virulence factors, such as LAM, PGL-I, and lipid metabolism (9, 10). Future studies should also address the possible advantage of combining DC-based IT with ICP blockade or other therapeutic approaches, such as antimicrobial and anti-inflammatory drugs.

## Concluding Remarks and Perspectives

**Figure 2:**
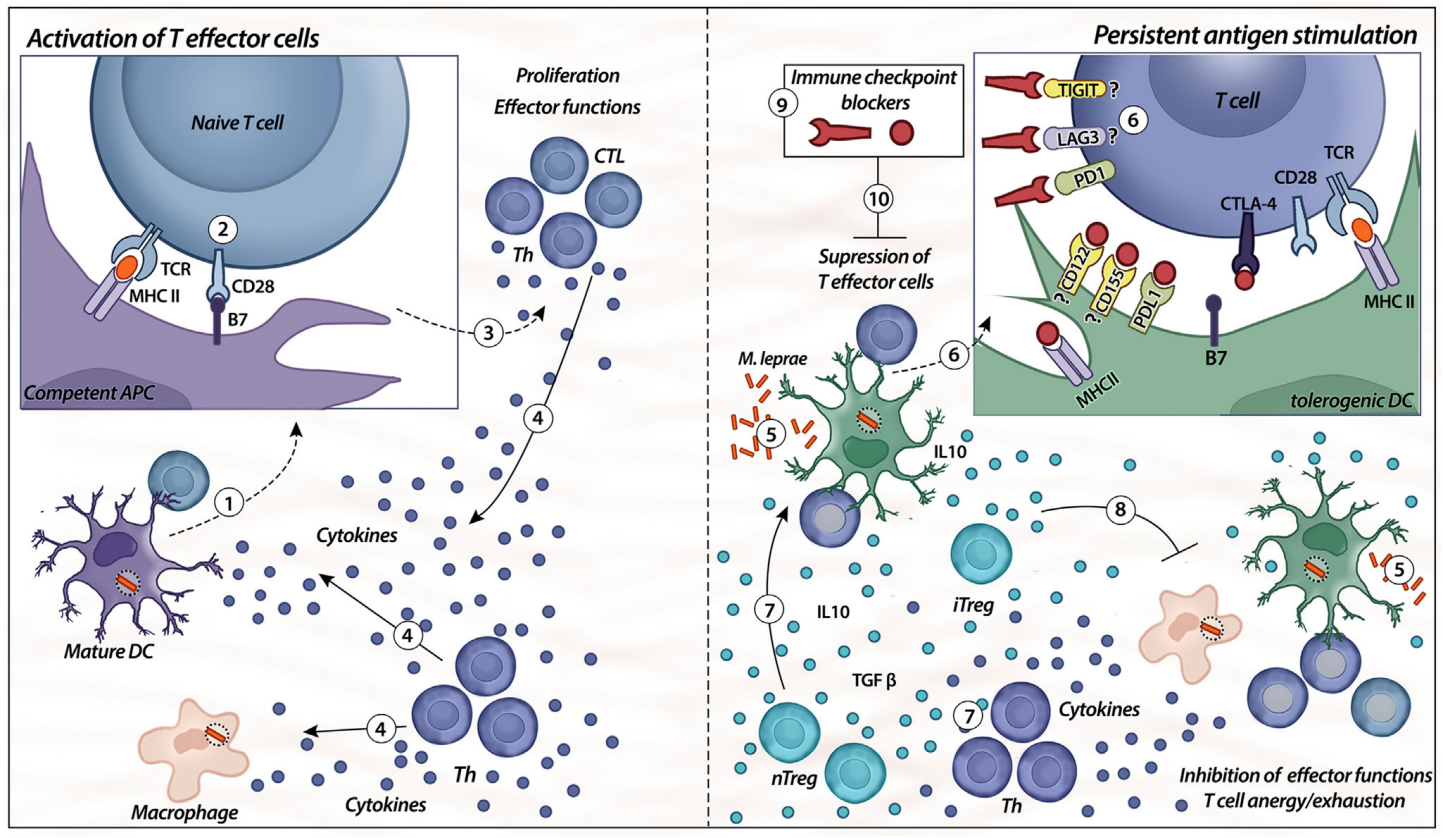
Immune checkpoints. Activation of T effector cells is initiated with competent/mature antigen-presenting cells (APCs), such as mature dendritic cells (DC) (1, 2). For the first signal, APC displays the antigen to the naïve T cell *via* a complex with MHC II on their surfaces that is recognized by TCR on the surface of T cells; the second signal is nonspecific, resulting from the binding of B7 ligand on the APC with its receptor, CD28, on the T cell (2). When both signals are provided (3), T cells (different types of T helper and CTLs) exert their effector functions, such as release of cytokines by different Th cells (IL-6, IL-2, IFN-γ, IL-12, and TNF-α) and cytotoxicity from CTL (4). The presence of chronic immune stimulation due to persistent microbial antigens impairs specific cellular immunity (5, 6). Expression of co-inhibitory molecules, such as PD-1, TIGIT, lymphocyte-activation gene-3 (LAG-3), and cytotoxic T-lymphocyte-associated protein 4 (CTLA-4), on lymphocytes and their respective ligands on the APC surface (PD-L1, CD122/155, MHC class II, and B7) induce specific T-cell anergy, leading to disseminated and progressive disease. In addition, there is higher differentiation of natural and induced types of Treg cells (nTreg/iTreg), as well as an imbalance of Th cells (7). The release of IL-10 and TGF-β from heterogeneous Treg cell subsets controls the immune response by the inhibition of effector functions, as well as induces tolerogenic phenotypes in DCs (8). The blockade of immune checkpoints, such as PD-1, CTLA-4, LAG-3, and TIGIT, might be a strategy to control the tolerogenic features observed in lepromatous leprosy patients (9, 10).

Altogether, evidence indicates that multiple factors are responsible for antigen-specific unresponsiveness in leprosy. We summarized some of these features and showed how ICP interfere with T-cell activation. We suggest that ICP blockade might interfere with leprosy pathogenesis (Figure 2). In our opinion, leprosy has been shown to have many interesting features concerning regulation that can be explored to better understand immunological mechanisms (11).

Immune checkpoint blockade has been widely applied in oncology as an adjuvant to chemotherapy and radiotherapy. In infectious diseases, ICP blockade is still a recent approach. In leprosy, it is even more critical because it is a neglected disease and probably ICP blockade might not be used as a large-scale therapy. There are some different strategies that can be used to achieve better treatment outcomes and improve the cellular response against *M. leprae*. In this context, BCG (re)vaccination for LL patients has been fulfilled without predictive results (79). For refractory patients, IT might be an additional strategy to control chronic disabilities.

Despite these promising results, IT based on ICP blockade has been associated with autoimmune and inflammatory events, such as oral mucositis and hepatitis (80, 81). In leprosy, increased immune stimulation has been associated with reactional states in LL patients and might be a disadvantage of this therapy. Certainly, more studies and clinical trials are needed to determine the role of Treg cells, ICPs and DCs as therapeutic targets to control *M. leprae* and leprosy progression.

## Ethics Statement

The experimental protocol used was approved by Ethical Committee of Bauru School of Dentistry, University of São Paulo and acquainted by Lauro of Souza Lima Institute Research Ethics Committee (protocol #148/2009). All subjects assigned informed consent that was obtained before performing the studies.

## Author Contributions

Conception and design: AC, JS, and GG. Development of methodology and formal analysis: HL, TG, TM, VM, MV, MN, FS, EM, and JB. Acquisition of data (provided animals, provided facilities, etc.): AC, JS, VS, and GG. Analysis and interpretation of data (e.g., statistical analysis) and writing of original draft: HL. Writing, review, and/or editing: HL, TG, VS, and AC. Funding acquisition and study supervision: AC.

## Conflict of Interest Statement

The authors declare that the research was conducted in the absence of any commercial or financial relationships that could be construed as a potential conflict of interest.

## Footnotes

^1^Peripheral mononuclear blood cells were obtained from untreated patients diagnosed with leprosy at the Dermatology Clinic of the Lauro de Souza Lima (ILSL) in Bauru, São Paulo, Brazil. The experimental protocol used was approved by Ethical Comitee of Bauru School of Dentistry of Bauru (protocol number #148/2009), University of São Paulo, and informed, and written consent was obtained from all subjects before performing the studies.
